# Syringaresinol alleviates acetaminophen-induced hepatocyte ferroptosis through the Nrf2/HO-1 pathway by targeting Caveolin-1

**DOI:** 10.1186/s13020-026-01421-0

**Published:** 2026-05-21

**Authors:** Lei Cao, Jiawei Zhang, Peng Song, Jia Zhao, Chuntao Wang, Yanhong Liu, Junhao Zang, Ziang Chen, Yang Gao, Wencong Tian, Zhi Qi

**Affiliations:** 1 School of Medicine, Nankai University, Tianjin, 300071 China; 2 Department of General Surgery, Tianjin Union Medical Center, The First Affiliated Hospital of Nankai University, Nankai University, Tianjin, 300121 China

**Keywords:** Syringaresinol, Acetaminophen, Ferroptosis, Caveolin-1, Nrf2/HO-1 pathway

## Abstract

Ferroptosis is reported to be closely involved in acetaminophen (APAP) overdose induced liver injury. The development of hepatoprotective drugs that target ferroptosis inhibition is highly valuable. In our previous work, we have confirmed the antioxidant properties of syringaresinol (SYR), but its effects on ferroptosis and the potential molecular mechanisms are unclear. This study aimed to investigate the impact of SYR on APAP-induced hepatocyte ferroptosis and elucidate the role of Caveolin-1 (CAV-1) in the protective function of SYR, focusing on its regulation of the Nrf2/HO-1 signaling pathway. In vitro validation was performed in APAP-treated AML12 and HL7702 cells. A mouse model of acute liver injury was established using intraperitoneal APAP injection. SYR was administered at low and high doses. We found that SYR significantly reduced ROS accumulation and Fe^2+^ overload, thereby alleviating APAP-induced cell death in vitro. Similarly, SYR alleviated APAP-induced liver injury by decreasing the level of Fe^2+^ and lipid peroxidation in mice. Western blot analysis also demonstrated SYR’s suppression of APAP-induced ferroptosis. Furthermore, SYR treatment dramatically elevated the protein expression of CAV-1 and activated the Nrf2/HO-1 pathway. Most importantly, knockdown of CAV-1 markedly abolished the protective effects of SYR against APAP-induced hepatocyte ferroptosis, as well as its activation effect on Nrf2/HO-1 pathway, indicating its critical role in SYR’s function on ferroptosis. Collectively, our results indicate that SYR protects against APAP-induced hepatocyte ferroptosis through the CAV-1/Nrf2/HO-1 signaling pathway.

**Graphical abstract:**

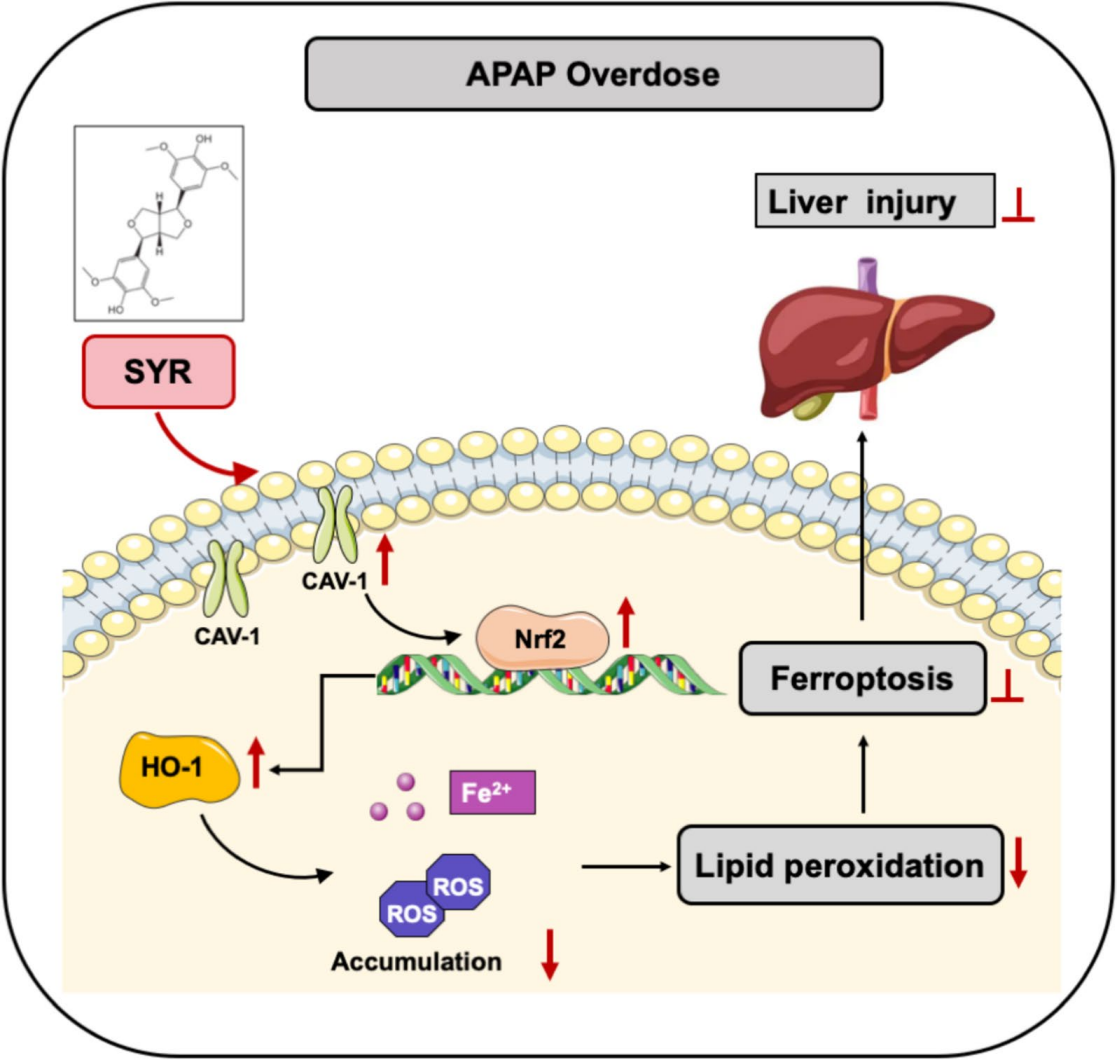

## Background

Acetaminophen (APAP) overdose is one of the main causes of liver-related death worldwide and has gained increasing attention in recent years [1]. Ferroptosis is a novel type of programmed cell death mechanism that requires the redox-active metal iron and is characterized by significantly elevated membrane lipid peroxidation [2, 3]. Yamada et al. reported that inhibition of ferroptosis can effectively prevent APAP-induced liver injury [4, 5], however, the underlying mechanisms remain incompletely elucidated. The identification of novel pharmacological agents or therapeutic targets aimed at ferroptosis inhibition is of paramount importance.

Syringaresinol (SYR, C22H26O8, PubChem CID: 100067) is a phytochemical constituent belonging to the lignan class and can be extracted from various parts of plants, such as the genus Syringa, Oleaceae and Fabaceae [6, 7]. In our previous work, we reported that SYR alleviated early diabetic retinopathy and protected against diabetic nephropathy by activating antioxidant pathways [8, 9]. Oxidative stress is considered a contributing factor to APAP-induced liver injury, nevertheless, the effects of SYR on ferroptosis or liver injury have not been reported. As the nuclear factor erythroid 2 (NF-E2)-related factor 2 (Nrf2)/heme oxygenase-1 (HO-1) signaling pathway has been demonstrated to participate in the adaptive response to oxidative stress, it is considered a viable drug target for various diseases [10, 11]. During the process of APAP-induced liver injury, whether SYR can exert protective effects through the Nrf2/HO-1 pathway still needs further investigation.

Caveolin-1 (CAV-1), a structural protein of the plasma membrane caveolae, is relatively ubiquitously present in all tissues and orchestrates a wide array of cellular activities including signal transduction and lipid transport [12]. Growing evidence shows that the upregulation of CAV-1 expression is implicated in the mitigation of a variety of liver-related diseases [13, 14], and CAV-1 has been confirmed to exert a protective effect against vascular injury by inhibiting oxidative stress and inflammation through the PKC/MAPK pathway [15], and promoting radioresistance in rhabdomyosarcoma through increased oxidative stress protection and DNA repair [16], thus we speculated that CAV-1 might perform a protective function against oxidative stress-induced liver damage. In the present study, we investigated the ability of SYR to suppress APAP-induced hepatocyte ferroptosis and further clarified the underlying molecular mechanisms, including the possible involvement of CAV-1 and the Nrf2/HO-1 pathway and their regulatory relationship.

## Materials and methods

### Animal model

Male C57BL/6J mice (aged 6–8 weeks) were purchased from Sibeifu Biotechnology (Beijing, China). All animal experiments were approved by the Medicine Ethical Committee of Institute of Radiation Medicine, Chinese Academy of Medical Sciences and Peking Union Medical College (Approval No. IRM/2-IACUC-2505-002).

SYR (≥ 98% purity by HPLC) was purchased from DASF Bio-Technology (Nanjing, China). Four groups were established: (1) the control (CON) group, (2) the APAP group, (3) the SYR25 group and (4) the SYR50 group. The mice in the SYR25 and SYR50 groups were administered SYR at a dose of 25 mg or 50 mg/kg body weight respectively by gavage every other day for 2 weeks, whereas those in the CON group and APAP group received an equal volume of vehicle. On the 15th day, the mice in the APAP, SYR25 and SYR50 groups were intraperitoneally injected with 300 mg/kg of APAP. After 12 h of APAP treatment, blood and liver tissues were collected.

### Histological assessments

Liver samples were paraffin-embedded and sectioned at a thickness of 4 μm. Hematoxylin and eosin (H&E) staining and immunohistochemical (IHC) staining were performed routinely [17]. For IHC analysis, the tissue sections were incubated with primary antibodies against CAV-1, Nrf2 or HO-1 overnight at 4 °C, followed by incubation with HRP-conjugated secondary antibodies and diaminobenzidine (DAB) substrate. Antibodies against CAV-1 and HO-1 were obtained from Proteintech Bio-Technology (Wuhan, China), and antibody against Nrf2 was purchased from Sigma-Aldrich (MO, USA).

### Biochemical assays

Aspartate aminotransferase (AST) and alanine aminotransferase (ALT) activities in the serum were assessed using assay kits from Nanjing Jian Cheng (Jiangsu, China). The levels of malondialdehyde (MDA), and the ratio of GSH (reduced glutathione)/GSSG (oxidized glutathione disulfide) in liver tissues and cell lines were determined with a Lipid Peroxidation MDA Assay Kit or GSH and GSSG Assay Kit (Beyotime Biotechnology, Shanghai, China). A Ferrous Ion Assay Kit was bought from Yuanye Bio-Technology (Shanghai, China) and used to examine the Fe^2+^ concentration. All the measurements were conducted following the manufacturer’s instructions.

### Examination of reactive oxygen species (ROS) production

Fresh tissues were embedded in OCT solution and sectioned at a thickness of 6 µm. The frozen sections were washed three times with PBS, followed by DHE staining (Bestbio Bio-Technology, Shanghai, China) for 60 min. And the ROS Assay Kit (Solarbio Science & Technology Co., Beijing, China) was used to measure ROS generation in AML and HL7702 cells. Images were acquired using a fluorescence microscope and quantified using the Image J software.

### Cell culture and drug treatment

AML12 cells (Procell Life Science & Technology, Wuhan, China) and HL7702 cells (BeNa Culture Collection, Xinyang, China) were seeded into cell culture plates and divided into the CON, APAP and SYR groups. The cells in the SYR group were preincubated with SYR (10 μmol/L, 24 h) and subsequently stimulated with SYR plus APAP (24 h). In the APAP group, cells were treated with APAP for 24 h.

### Assays of cell viability

CCK-8 analysis (Abbkine Scientific Co., Ltd, Wuhan, China) and Acridine Orange/Ethidium Bromide (AO/EB) staining (Yuanye Bio-Technology, Shanghai, China) were performed to assess the cell viability. The AO/EB working solution was prepared at the following ratio: reagent (A): reagent (B): reagent (C): PBS = 1: 1: 8: 990. After being washed three times with PBS, 0.5 mL of the working solution was added to each well of the cell plates and incubated at room temperature in the dark for 5–15 min.

### Small interfering RNA (siRNA) transfection

Mouse/human siRNAs targeting CAV-1 were designed and acquired from OBIO Biotech Co., Ltd, Shanghai, China, and transfection was performed as described previously [16]. In brief, cells were seeded in six-well plates, and transfected with these siRNAs using Lipo6000 reagent (Beyotime Biotechnology, Shanghai, China). The plates were incubated at 37 °C, and the protein levels were determined after 48 h.

### Western blot

The western blotting analysis was performed following the previously mentioned procedures [18]. Protein samples were separated by 11% SDS-PAGE and transferred onto 0.2 μm PVDF membranes. After being blocked with 5% nonfat milk powder, the membranes were incubated with the specific primary antibodies. HRP-conjugated secondary antibodies were subsequently used for detection, and the immunoreactive signals were analyzed using an Allcam detection system (Tanon, Canton, MA, USA). Antibodies against glutathione peroxidase 4 (GPX4) and β-tubulin were purchased from Selleck Chemicals (TX, USA), and antibodies against ferritin light-chain (FLC) and ferritin heavy-chain (FHC) were bought from ABclonal Technology (Wuhan, China). Antibody against xCT was obtained from Abcam (CB, UK).

### Statistical analysis

Data were presented as the mean ± SD. Statistical significance was analyzed using one-way ANOVA followed by multiple comparisons. *P* < 0.05 was considered statistically significant.

## Results

### SYR prevented APAP-induced cell death in vitro by inhibiting ferroptosis

**Fig. 1:**
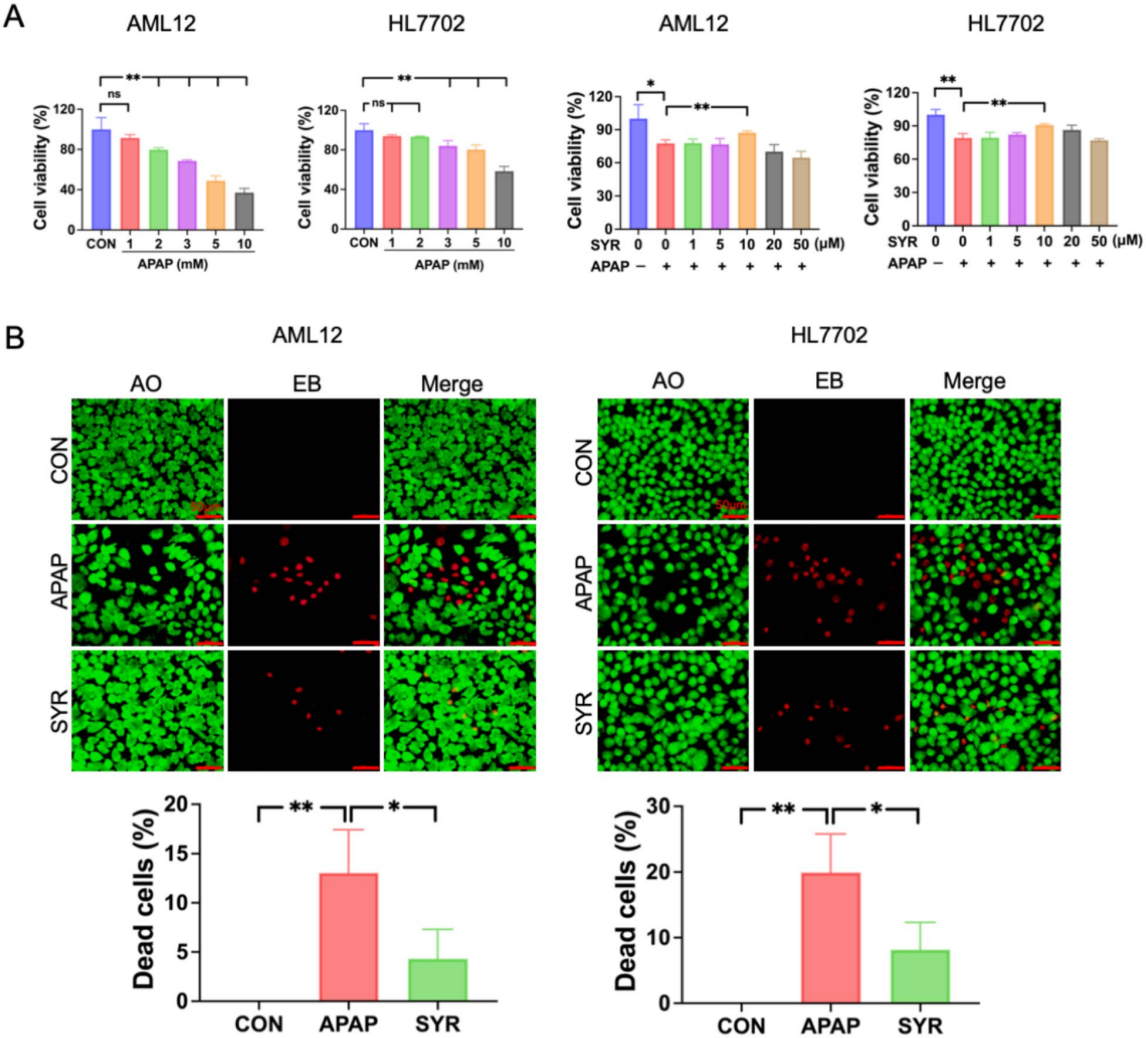
Effects of SYR and APAP on cell viability. (**A**) CCK-8 and (**B**) AO/EB fluorescence staining were performed to determine the cell survival (n = 3). The red line represented a scale bar of 50 μm. Values are expressed as mean ± SD. *p < 0.05, **p < 0.01

To assess the cytotoxicity of APAP, AML12 and HL7702 cells were exposed to different concentrations of APAP (1 mM, 2 mM, 3 mM, 5 mM and 10 mM), and the cell viability decreased in a dose-dependent manner (Fig. 1A). Specifically, 1 mM APAP treatment did not result in detectable cytotoxicity in AML12 cells, whereas exposure to all the other APAP concentrations led to significant reductions in cell viability. In HL7702 cells, 3 mM, 5 mM and 10 mM treatment of APAP dramatically induced cytotoxicity, while both the 1 mM and 2 mM APAP groups presented cell viability comparable to that of CON group. On the basis of the above results, 2 mM and 3 mM were selected for the treatment of AML12 and HL7702 cells, respectively, in the subsequent experiments. To assess the protective effects of SYR in vitro, cells were exposed to APAP conditions alongside five varying concentrations of SYR (1 μM, 5 μM, 10 μM, 20 μM and 50 μM). As shown in Fig. 1A, 10 μM SYR notably reduced APAP-induced cell death in both cell lines. In agreement with these findings, AO/EB staining showed that in the two cell lines, APAP treatment resulted in cell death rate of approximately 13% and 20% respectively, while the rates in SYR groups were approximately half that of the APAP group. These results demonstrated that APAP treatment substantially increased the proportion of dead cells relative to that in the CON group, whereas SYR treatment decreased the ratio of dead cells to total cells in both the AML12 and HL7702 models (Fig. 1B).

**Fig. 2:**
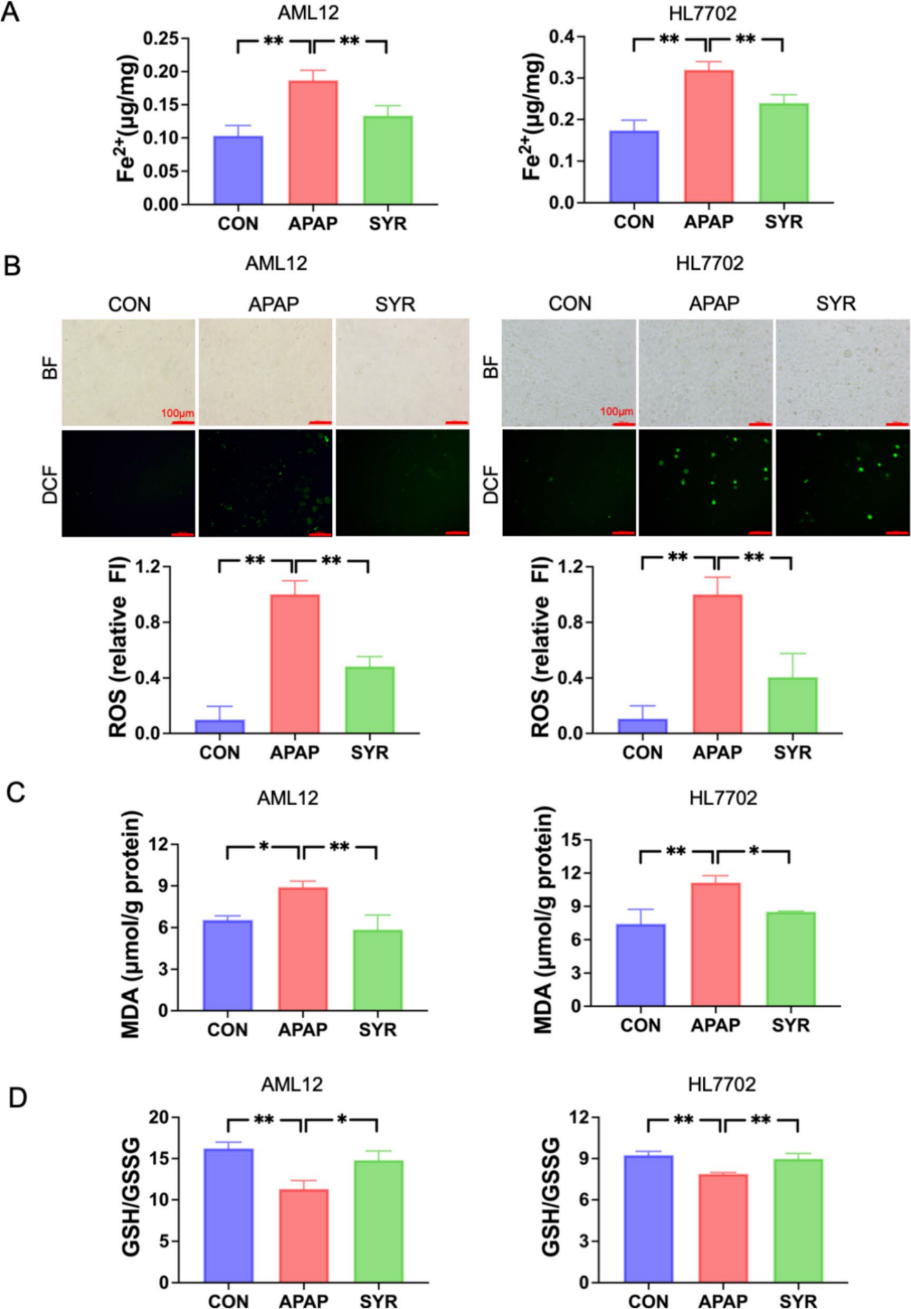
Effects of SYR on APAP-induced ferroptosis. Changes of the levels of **A** Fe^2+^, **B** ROS and **C** MDA after SYR treatment (n = 3). **D** The ratio of GSH to GSSG was examined (n = 3). The red line represented a scale bar of 100 μm. Values are expressed as mean ± SD. *p < 0.05, **p < 0.01

**Fig. 3:**
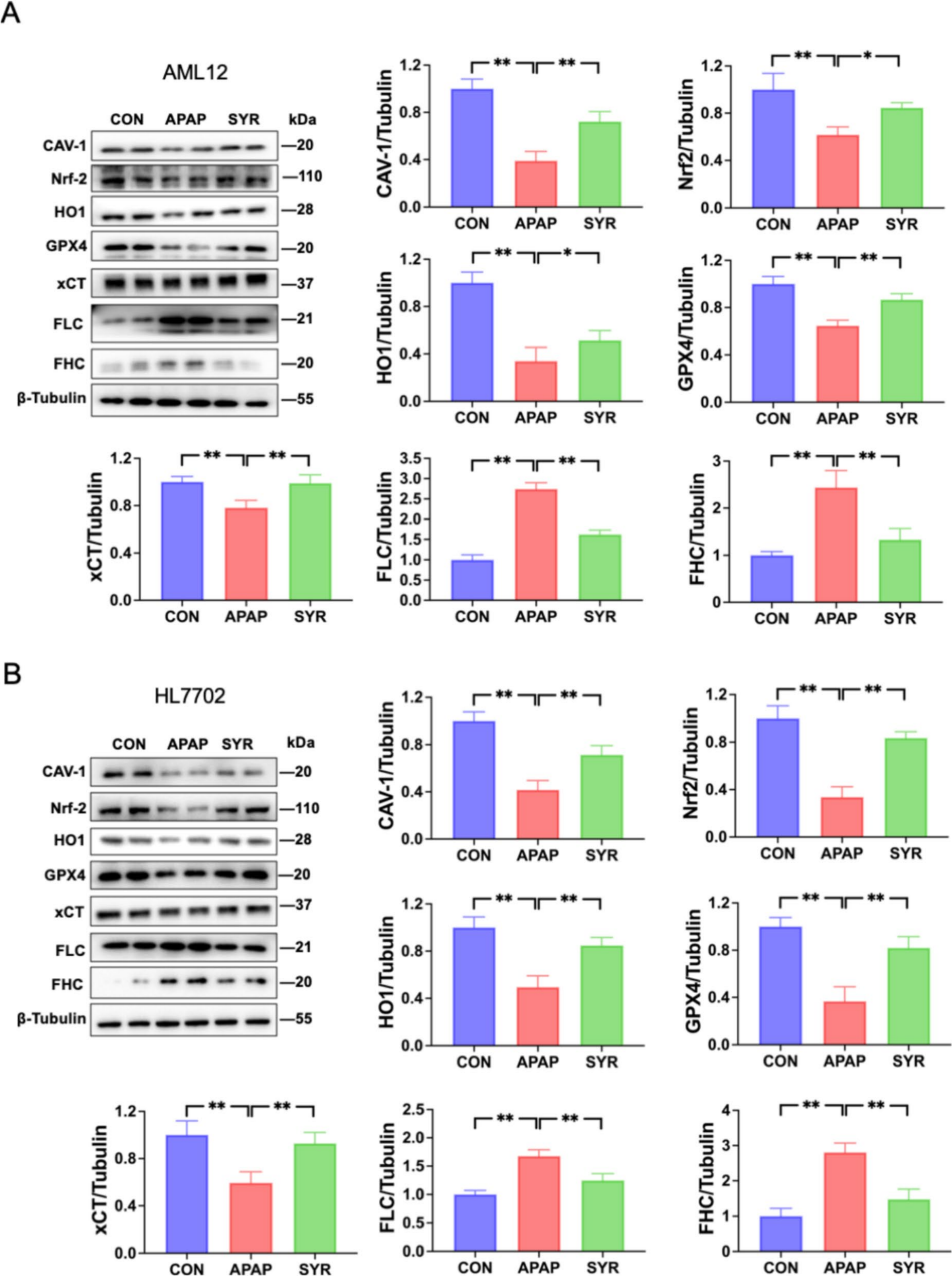
Regulation of SYR on CAV-1 expression and the Nrf2/HO-1 pathway. Protein levels of CAV-1, Nrf2, HO-1 and the ferroptosis associated proteins in **A** AML12, **B** HL7702 cells were detected by western blot (n = 6). The data are expressed as the mean ± SD. *p < 0.05, **p < 0.01

Given that ferroptosis is a critical mechanism in APAP’s hepatotoxicity, we next investigated the impact of SYR on ferroptosis. Biochemical assays showed that the levels of Fe^2+^, ROS and MDA were significantly elevated following APAP treatment, whereas SYR administration notably attenuated their concentrations in both AML12 and HL7702 cells (Fig. 2A–C). Additionally, examination of the GSH/GSSG ratio revealed a marked reduction in the ratio of GSH to GSSG in the APAP group, whereas the ratio was substantially increased by SYR treatment (Fig. 2D). Furthermore, western blotting analysis revealed a pronounced decrease in the levels of GPX4 and xCT expression, along with a significant increase in the protein levels of FLC and FHC following APAP stimulation. Notably, the changes in these molecules were markedly reversed following SYR administration (Fig. 3). Taken together, these findings suggest that SYR protects against APAP-mediated hepatotoxicity, at least in part, through the inhibition of ferroptosis.

### SYR treatment enhanced the expression of CAV-1 and activated the Nrf2/HO-1 pathway

To ascertain whether CAV-1 serves as a pivotal target of SYR, its expression was examined by western blotting. The results demonstrated a market reduction in the CAV-1 expression in cells of APAP group, which was significantly restored upon SYR treatment (Fig. 3). The Nrf2/HO-1 signaling pathway plays a crucial role in the response to oxidative stress, thereby participating in the regulation of ferroptosis in a variety of diseases. Western blot analysis confirmed that APAP stimulation caused a significant down-regulation in the protein levels of Nrf2 and HO-1, which were elevated by SYR treatment (Fig. 3).

### SYR treatment protects mice against APAP-induced liver injury through the inhibition of ferroptosis

**Fig. 4:**
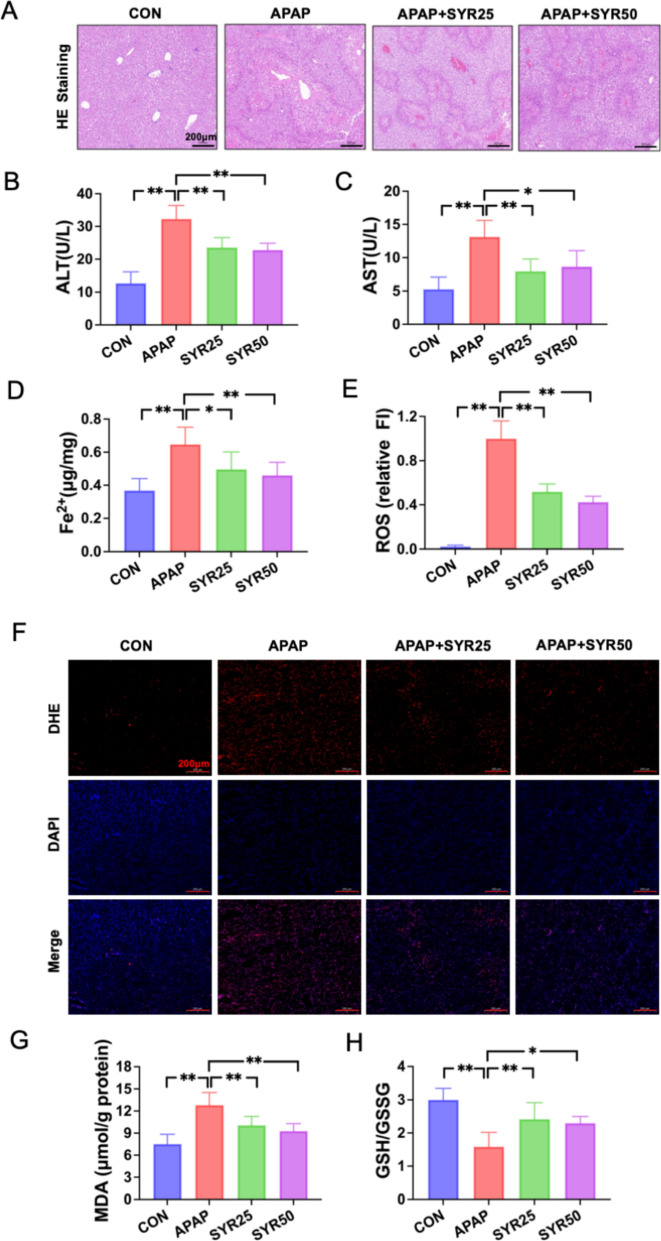
SYR attenuated APAP-induced hepatocyte ferroptosis in mice. **A** H&E staining was performed to assess liver injury after APAP injection (scale bar = 200 μm). Serum **B** ALT and **C** AST levels were measured in mice (n = 6). The levels of **D** Fe^2+^, **E**, **F** ROS, **G** MDA, and **H** the ratio of GSH to GSSG were examined in liver tissues (n = 6). The red line represented a scale bar of 50 μm. The data are expressed as the mean ± SD. *p < 0.05, **p < 0.01

**Fig. 5:**
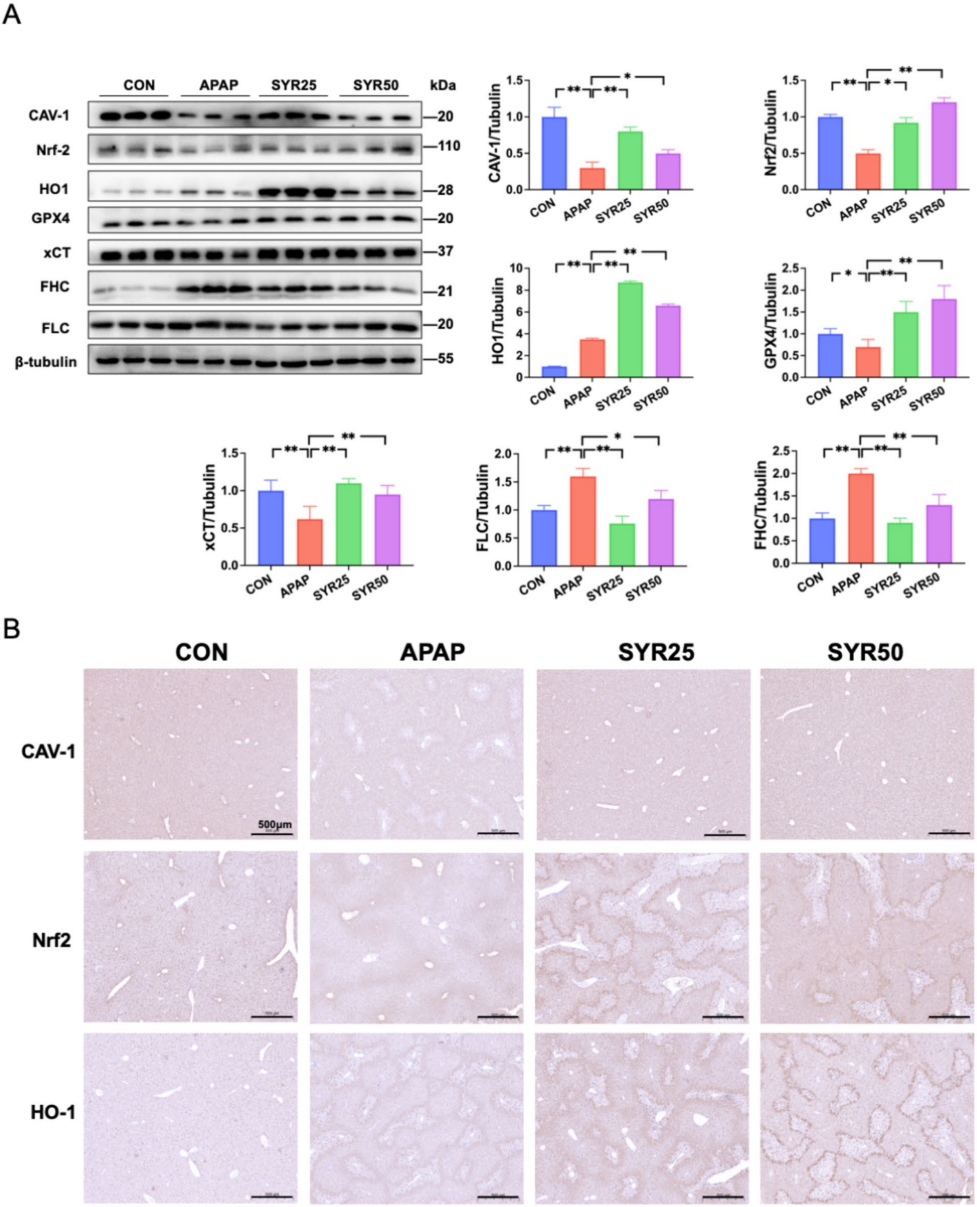
Changes of CAV-1 expression and the Nrf2/HO-1 pathway in mice following SYR administration. **A** Expression of CAV-1, Nrf2, HO-1 and the ferroptosis associated proteins in liver tissues was detected by western blot (n = 6). The data are expressed as the mean ± SD. **B** The levels of CAV-1, Nrf2 and HO-1 were measured by IHC staining (scale bar = 500 μm)

We administered SYR (25 mg/kg and 50 mg/kg body weight) to the mice for 2 weeks before APAP injection to evaluate its effects on APAP-induced liver injury. H&E staining demonstrated that the SYR administration alleviated histopathological damage to the liver at both dosing concentrations, as shown in Fig. 4A. Correspondingly, significant increases in the serum ALT and AST levels were observed following APAP treatment, reflecting impaired liver function, whereas SYR administration of the two doses significantly decreased their levels (Fig. 4B, C). To further assess SYR’s impact on ferroptosis, the levels of Fe^2+^, ROS and MDA in liver tissues were examined. Notably, these indicators were enhanced in APAP treated mice relative to the CON group, whereas SYR treatment significantly down-regulated their accumulation (Fig. 4D–G). In parallel, the results of GSH assay showed that liver tissues from APAP group displayed a marked decline in the ratio of GSH to GSSG compared to CON group. Nevertheless, the ratio was substantially restored in both the SYR25 and SYR50 groups (Fig. 4H). Furthermore, western blotting further revealed decreased liver expression of GPX4 and xCT, along with enhanced protein levels of FLC and FHC after APAP challenge, which was consistent with the results of in vitro experiments. Similarly, the changes in these molecules were significantly reversed following SYR administration (Fig. 5A). Collectively, these findings indicate that SYR effectively protects against APAP-induced liver injury through the inhibition of ferroptosis in mice.

### SYR-treated mice displayed enhanced protein expression of CAV-1, Nrf2 and HO-1 in liver

Western blotting and IHC staining were performed to ascertain the involvement of CAV-1, Nrf2 and HO-1 in SYR’s protective effect. Compared with the CON group, APAP stimulation markedly decreased the expression of CAV-1 and Nrf2 in liver, whereas SYR treatment significantly promoted the expression of these two molecules (Fig. 5A, B). Of note, the amount of HO-1 in livers of the mice from CON group was at a relatively low level, and the expression of HO-1 in the APAP-treated mice was increased to about 3.5 times, which differed from the results of the in vitro experiments. However, liver tissues from the two doses of SYR groups (25 mg/kg and 50 mg/kg) displayed a further significant increase in HO-1 expression when compared to APAP-treated mice, and this trend was consistent with the results in the cells lines. In summary, these results indicate that SYR up-regulated CAV-1 expression and activated the Nrf2/HO-1 pathway, and thereby mitigating APAP-induced ferroptosis in the mouse liver.

### Interference of CAV-1 abolished the protective effect of SYR against APAP-induced ferroptosis in vitro

**Fig. 6:**
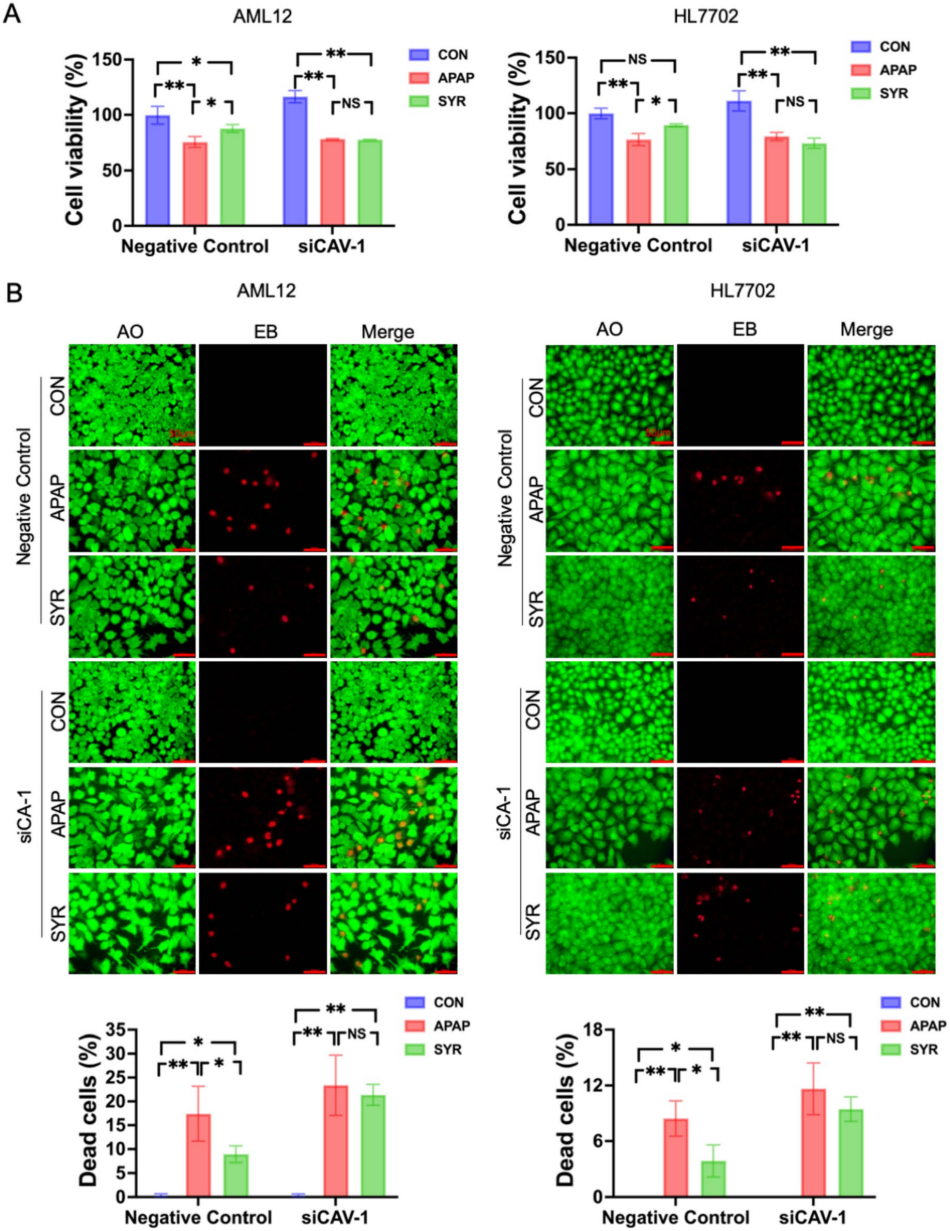
Effects of SYR on cell viability after CAV-1 was knocked-down. **A** CCK-8 assay and **B** AO/EB fluorescence staining were performed to determine the cell survival (n = 3). The red line represented a scale bar of 50 μm. Values are expressed as mean ± SD. *p < 0.05, **p < 0.01

**Fig. 7:**
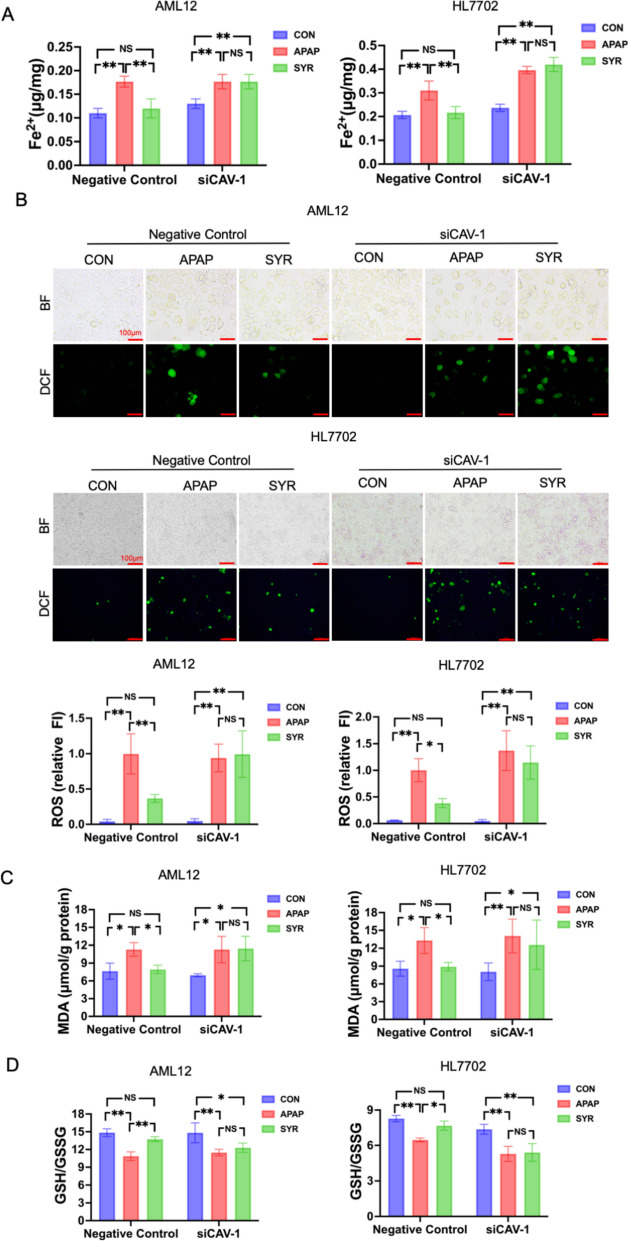
Interference of CAV-1 abolished the inhibitory effect of SYR on APAP-induced ferroptosis (n = 3). The levels of **A** Fe^2+^, **B** ROS, **C** MDA and **D** the ratio of GSH/GSSG were examined (n = 3). The red line represented a scale bar of 100 μm. Values are expressed as mean ± SD. *p < 0.05, **p < 0.01

**Fig. 8:**
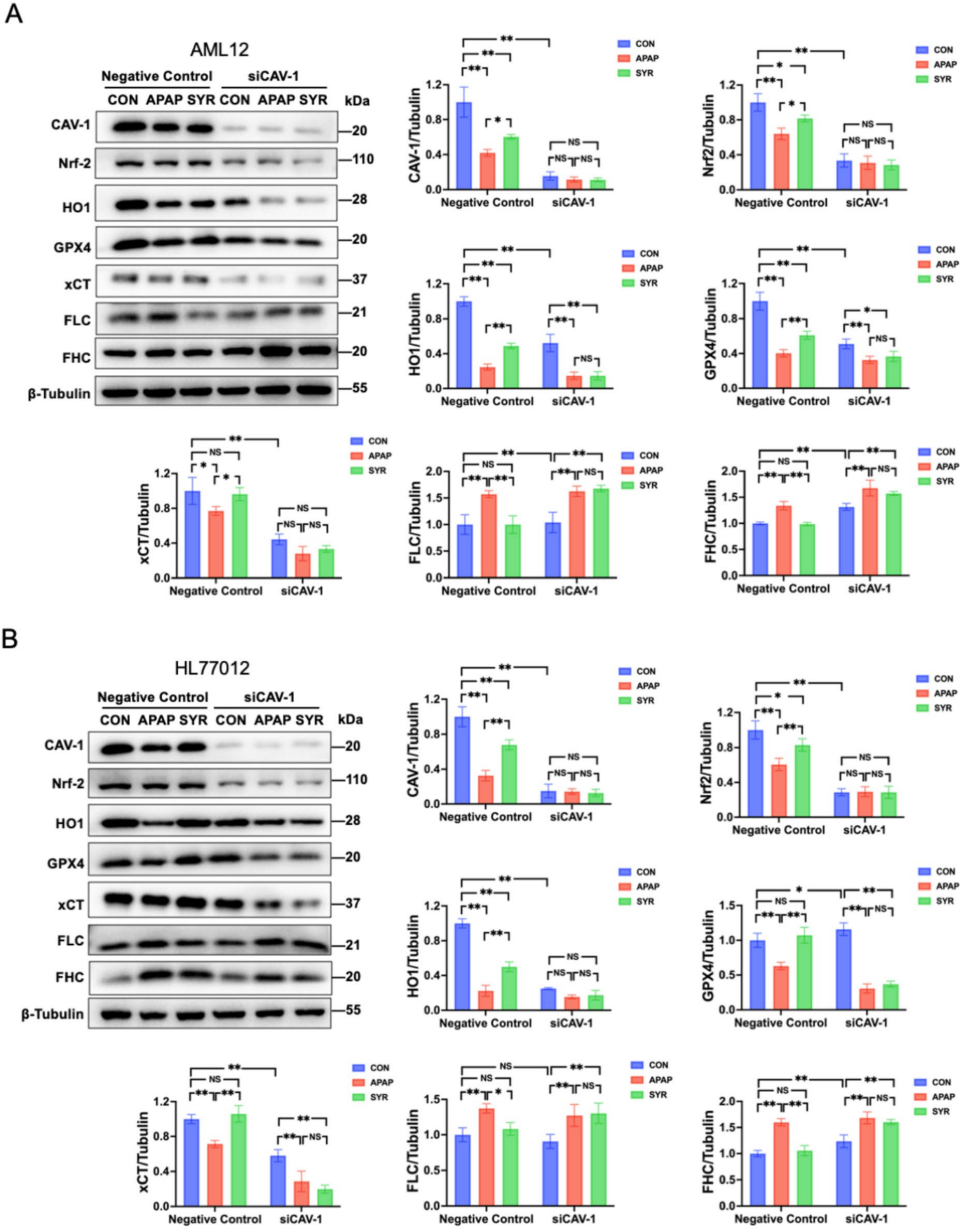
SYR failed to activate the Nrf2/HO-1 pathway when CAV-1 was knocked down. The expression of the aforementioned molecules in **A** AML12 and **B** HL7702 cells after siRNAs treatment (n = 3). Values are expressed as mean ± SD. *p < 0.05, **p < 0.01

To further elucidate the underlying molecular mechanisms, siRNAs targeting CAV-1 were employed to negate the influence of SYR on CAV-1 expression in AML12 and HL7702 cells. As illustrated in Fig. 8, siRNA transfection showed a high inhibitory efficiency, significantly deceasing CAV-1 protein expression. And importantly, in the groups of Negative Control, SYR treatment substantially increased cell viability and reduced the proportion of dead cells following APAP stimulation. However, in the groups of siRNA, the cytotoxicity of combined administration of SYR and APAP was comparable to that of APAP treatment (Fig. 6). In addition, in the Negative Control cells of AML12 and HL7702, compared with the CON group, the Fe^2+^ levels in the APAP group were approximately 1.8 times and 1.5 times higher, respectively, while the levels in the SYR group were comparable to those in the CON group, and were significantly lower than those in the APAP group. In the siCAV-1 cells, the Fe^2+^ levels in both the APAP and SYR treatment groups were significantly increased, and there was no significant statistical difference between these two groups (Fig. 7A). Furthermore, in Negative Control cells, SYR treatment markedly downregulated the levels of ROS and MDA, and significantly increased the ratio of GSH to GSSG relative to the APAP group. However, in cells with CAV-1 silencing, the SYR group did not present significant alterations in the levels of these indicators compared to the APAP group (Fig. 7B–D).

We further investigated the protein levels of ferroptosis associated molecules and Nrf2/HO-1 pathway. The results demonstrated that the expression of Nrf2, HO-1, GPX4 and xCT was significantly elevated in the Negative Control: SYR group relative to the Negative Control:

CON group, whereas FLC and FHC were decreased in the former group. However, CAV-1 depletion dramatically abrogated the regulation of the afore-mentioned proteins by SYR, indicating that SYR failed to activate the Nrf2/HO-1 signaling pathway and inhibit APAP-induced ferroptosis when CAV-1 was silenced (Fig. 8). Collectively, these results suggest that CAV-1 is essential for the protective effect of SYR against APAP-induced ferroptosis by activation of Nrf2/HO-1 signaling pathway.

## Discussion

APAP overdose is the number one cause of acute liver failure in the U.S. and the UK, and epidemiological studies from the U.S. show that almost half of APAP overdoses are unintentional [19]. Large amounts of N-acetyl-p-benzoquinone imine (NAPQI) are rapidly generated and overwhelm the GSH stores in the liver when APAP is taken as an overdose. Subsequently NAPQI reacts with cysteine groups on proteins, forming protein adducts [20], which is a hallmark of APAP pathophysiology. Generally, oxidative stress is identified as the core mechanism of APAP-induced acute liver injury [20]. At present, N-acetyl cysteine (NAC), a known antioxidant, is recommended as the only therapeutic option for APAP-overdosed patients, however, its clinical use is limited by the adverse effects and narrow therapeutic window [21], highlighting the demand for the development of safer and more effective drugs. SYR has been reported to possess potent antioxidant functions, but it’s impact on APAP-induced hepatotoxicity has not yet been investigated. In this study, we demonstrated for the first time that SYR alleviates APAP-induced liver injury through the Nrf2/HO-1 pathway by targeting Caveolin-1.

In the case of severe impairment of antioxidant defense mechanisms with APAP, ferroptosis pathway is activated and contributes to hepatocyte death [19, 22]. As one form of programmed cell death that has recently come into focus, ferroptosis is regulated mainly by iron homeostasis, lipid metabolism, and GSH-dependent redox balance, a key feature of ferroptosis is the extensive accumulation of lipid peroxides [23]. Ferritin is composed of two subunits, namely ferritin heavy chain (FHC) and ferritin light chain (FLC) [24]. As an iron storage protein within cells, its degradation leads to excessive release of labile Fe^2+^ into the cytoplasm, promoting the production of hydroxyl and hydroperoxyl radicals through a Fenton-type reaction, which is an indispensable driver of ferroptosis [25]. The status of solute carrier family 7 member 11 (SLC7A11; also known as xCT) is closely involved in production of GSH [26]. Since the iron-dependent lipid peroxidation will be facilitated by depletion of GSH, it is identified as a critical mediator for sensitivity to ferroptosis [27]. In addition, glutathione peroxidase 4 (GPX4) is responsible for decreasing lipid hydroperoxides using reducing equivalents from GSH, and ferroptosis is typically aggravated when GPX4 is inhibited. In the present study, APAP treatment markedly elevated the levels of Fe^2+^ and protein expression of FHC and FLC, alongside with a significant reduction in protein levels of GPX4 and xCT, as validated in both in vivo and in vitro models. The protective effect of inhibiting ferroptosis against APAP-induced liver injury has been confirmed by accumulating evidence. For instance, Shi et al. [5] demonstrated that activation of the Nrf2/GSH axis antagonizes ferroptosis, thereby protecting APAP-induced liver injury. Similarly, Li et al. [28] reported that activated Nrf2 signaling modulates ferroptosis process, exerting significant effects on hepatotoxicity of APAP. Furthermore, Niu et al*.* synthesized ultrasmall poly(acrylic) acid coated Mn3O4 nano-particles (PAA@Mn3O4-NPs, PMO), which could potently suppress ferroptosis. Subsequently, they injected PMO into mice and observed a significant protective effect in acute liver injury [29]. These research findings highlight that targeting ferroptosis may represent a promising therapeutic strategy to mitigate liver toxicity caused by APAP overdose. Besides, it is well established that APAP-induced hepatotoxicity leads mainly to hepatocyte necrosis [19, 30]. Initially, the accumulation of the NAPQI protein in mitochondria leads to continuous oxidative stress and dysfunction, followed by gradual liver cell necrosis [30]. However, the main mode of cell death induced by APAP is still controversial, and different mechanisms of cell death may overlap with each other, more in-depth research is needed to explore the regulatory mechanisms involved in APAP-induced liver injury.

According to research, SYR showed slow absorption, extensive tissue distribution, and prolonged systemic exposure [6]. In pharmacokinetic studies of SYR by oral administration, the area under the curve extrapolated to infinity (AUC(0–∞)) ranged from 464.17 to 1873.12, the clearance rate (CL) ranged from 19.67 to 21.84 L/h/kg, the elimination half-life (t_1/2_) ranged from 4.81 to 6.07 h and the absolute oral bioavailability of was calculated at 7.69% [6]. SYR exhibits significant antioxidant properties due to the presence of methoxy electron-donating groups near phenolic hydroxyl neighborhood. It is now acknowledged for the beneficial effects in many diseases. Kim et al. [31] reported that SYR inhibited α-melanocyte-stimulating hormone-induced ROS generation and melanogenesis; Choi et al. [32] identified that SYR mitigated oxidative stress-induced skin aging through regulation of autophagy; in our previous work, we have demonstrated that SYR protected against diabetic nephropathy by activating antioxidant pathway [9]. As oxidative stress acts as a crucial trigger for ferroptosis, we hypothesized that SYR could effectively inhibit APAP-induced hepatocyte ferroptosis. As expected, in the present study, SYR decreased the intracellular concentration of Fe^2+^, as well as the expression of FLC and FHC in APAP treated mice or cell lines. Concurrently, SYR’s inhibition on oxidative stress and lipid peroxidation was also verified both in vivo and in vitro. These results provide important evidence for the anti-ferroptosis function of SYR. In addition, SYR has been shown to possess anti-inflammatory effects [33, 34], and inflammation is also recognized as one of the main mechanisms involved in the pathogenesis of APAP-induced hepatotoxicity [30, 35], however, it remains unclear whether the protective effect of SYR against APAP-induced liver injury is related to modulation of the inflammatory response. Lastly, it should be noted that the application prospects of monomer extracts of natural herb are not only evaluated by their effectiveness and low toxicity but also by their good pharmacokinetic characteristics. After fully clarifying the application potential of SYR, it is necessary to explore how to enhance its bioavailability.

Investigations of the role of CAV-1 in liver injury have mainly focused on non-alcoholic fatty liver disease (NAFLD). CAV-1 has been shown to ameliorate hepatic injury in by inhibiting ferroptosis via the NOX4/ROS/GPX4 pathway [13], alleviate APAP-induced vascular oxidative stress and inflammation [15], and ameliorate APAP-aggravated inflammatory damage and lipid deposition via the ROS/TXNIP/NLRP3 pathway [36]. Recently, a study led by Deng revealed that NAFLD hepatocytes exhibits decreased expression of CAV-1, accompanied by iron metabolism disorder. CAV-1 effectively enhanced the iron storage capacity of hepatocytes via activating the FLC/FHC pathway, resulting in suppression of the oxidative stress induced by excess ferrous ions [37]. Despite these findings, comprehensive exploration of the role of CAV-1 in liver diseases remain limited, necessitating further evidence to substantiate its therapeutic potential. We examined the expression of CAV-1 in the APAP- and SYR-treated mice in this study, and the results primarily proved that SYR reversed the down-regulation of CAV-1 caused by APAP treatment. More importantly, CAV-1 depletion abolished SYR’s inhibitory effect on APAP mediated ferroptosis. We found no significant changes in the levels of Fe^2+^, MDA and ROS, as well as the ratio of GSH/GSSG between APAP group and SYR group in CAV-1-knockdown cells. Western blot analysis further confirm that the regulation of SYR on expression of FLC, FHC, GPX4 and xCT was diminished following CAV-1 interference. Our study demonstrates that CAV-1 may serve as a potential target in the treatment of APAP-induced liver injury. CAV-1 is an oxidative stress-related protein, its downregulation under oxidative stress conditions is reported to be associated with a protein degradation mechanism via the ubiquitin–proteasome pathway [38]. Consistent with our results, Fu et al. reported that APAP reduced CAV-1 expression in liver [15, 39]. Although in many studies, including our current research, it has been demonstrated that CAV-1 plays a protective role in liver damage caused by various factors, there are also some literature suggesting that CAV-1 deficiency is helpful in alleviating some drug-induced liver injury [40–42]. Moreover, CAV-1 has well-documented dual roles, as both pro- and anti-ferroptotic, in different disease settings [43–45]. Different diseases may affect the distribution and signal transduction of CAV-1, and at different stages of disease progression, the expression of CAV-1 also undergoes dynamic changes, therefore, the relationship between CAV-1 expression and hepatocyte ferroptosis still needs to be studied in vivo and in vitro for different disease states.

The Nrf2/HO-1 signaling pathway was an important participant in defending against oxidative stress, and its activation can confer resistance to ferroptosis [46–48]. Consistently, we observed a significant down-regulation of expression of Nrf2 and HO-1 in APAP treated mice and cell lines; notably, this down-regulation was markedly reversed following treatment with SYR. Crucially, the application of siRNAs targeting CAV-1 abrogated the modulatory effects of SYR on Nrf2/HO-1 pathway in APAP treated cells, leading to severe ferroptosis. These results suggested Nrf2/HO-1 signaling pathway functions downstream of CAV-1. Of note, in vitro experiments showed that APAP decreased the expression of HO-1 compared to the control cells, while APAP treated mice displayed higher protein level of HO-1 relative to the normal. We hypothesized that under normal conditions, the oxidation system and the antioxidant system are in a balanced state, and the amount of ROS is limited in this context, therefore antioxidant enzymes, including HO-1, are expressed at a low level accordingly. APAP stimulation caused excessive production of ROS, and antioxidant system was upregulated in response to oxidative stress. However, the state of oxidative stress and the antioxidant system in cell lines are likely abnormal to gain the ability of immortality. The key focus of this study is on the effect of SYR on APAP-induced ferroptosis, and SYR treatment enhanced HO-1 expression both in the in vivo and in vitro experiments when compared to the APAP group.

## Conclusions

In summary, our study underscores the important role of CAV-1 in the anti-ferroptosis effect of SYR. The results demonstrates that SYR reverses the downregulation of CAV-1 expression observed during APAP-induced hepatocyte ferroptosis, thereby activating Nrf2/HO-1 signaling pathway and preventing excessive accumulation of ROS and lipid peroxidation. To the best of our knowledge, this is the first report linking SYR’s inhibitory effects on ferroptosis to CAV-1 expression. These findings provide novel mechanistic insights into the role of CAV-1 in ferroptosis and suggest that SYR may serve as a lead compound for the development of innovative therapeutic strategies for hepatoprotection.

## Data Availability

The datasets used and/or analysed during the current study are available from the corresponding author on reasonable request.

## Abbreviations

APAP: Acetaminophen
SYR: Syringaresinol
CAV-1: Caveolin-1
Nrf2: Nuclear factor erythroid 2 (NF-E2)-related factor 2
HO-1: Heme oxygenase-1
AST: Aspartate aminotransferase
ALT: Alanine aminotransferase
MDA: Malondialdehyde
GSH: Reduced glutathione
GSSG: Oxidized glutathione disulfide
ROS: Reactive oxygen species
GPX4: Glutathione peroxidase 4
FLC: Ferritin light chain
FHC: Ferritin heavy chain
NAPQI: N-acetyl-p-benzoquinone imine
NAFLD: Nonalcoholic fatty liver disease
